# Prevalence of glucose-6-phosphate dehydrogenase deficiency and its association with *Plasmodium falciparum* infection among children in Iganga distric in Uganda

**DOI:** 10.1186/1756-0500-7-372

**Published:** 2014-06-18

**Authors:** Denis Bwayo, Mark Kaddumukasa, Henry Ddungu, Fred Kironde

**Affiliations:** 1 Department of Medicine, Makerere University College of Health, Sciences, Kampala, Uganda; 2 Department of Biochemistry, Makerere University College of Health, Sciences, Kampala, Uganda

## Abstract

**Background:**

Glucose-6-phosphate dehydrogenase (G6PD) is a metabolic enzyme involved in the pentose phosphate pathway, its especially important in red blood cell metabolism. Glucose-6-phosphate dehydrogenase deficiency is an X-linked recessive hereditary disease characterised by abnormally low levels of G6PD. About 400 million people worldwide have a deficiency of this enzyme. The remarkable geographic correlation of G6PD deficiency distribution with historical endemicity patterns of malaria has led to suggestions that the two could be linked. Some studies have concluded that G6PD deficiency confers resistance to malaria.

**Objective:**

To determine the prevalence of G6PD deficiency, and determine its relationship with prevalence and incidence of *P. falciparum* infection among children in Uganda.

**Methods:**

This was longitudinal study involving 245 children, 135 were actively followed up for 12 months. G6PD status was assessed for using PCR-RFLP method. A thick smear was done to determine presence of plasmodium trophozoites and parasite densities.

**Results:**

A total of 245 children between 6 months and 9 years were recruited. Of these 46.5% were males. Overall prevalence for the X-linked G6PD A- mutation was; 79.59% wild type, 12.65% heterozygous and 7.76% homozygous or hemizygous. Among the males 14% were hemizygous. At baseline, 40.8% had asymptomatic *P falciparum* infection. There was no statistically significant difference in prevalence and incidence rates of *malaria* infection among the different G6PD genotypes with prevalence among heterozygous, homozygous, and wild type being 29%, 42.6% and 43% respectively (p = 0.11) and incidence among heterozygous and wild type being 0.56 and 0.52 episodes/year (p = 0.5). The heterozygous G6PD A- females had a lower parasite density compared to the wild type (2505 vs 941 parasites/μL; P = 0.024).

**Conclusions:**

This study showed that 20.41% of the population in this part of Uganda carry the G6PD A-mutation, within the range of 15-32% seen in other parts of Africa. *P. falciparum* infection incidence and prevalence rates are similar among the G6PD genotypes though, once infected, *P. falciparum* parasite densities are lowest among G6PD A- heterozygous females. This suggests differences in *P. falciparum* infection rates and severity of disease could be mediated by differences in parasite densities among the different G6PD genotypes.

## Background

Glucose-6-phosphate dehydrogenase (G6PD) is an enzyme that is present in the cytoplasm of all cells of the body. It plays a pivotal role in the generation of nicotinamide adenine di-phosphosphate-H (NADPH) in cells. G6PD is involved in the first step of the Pentose Phosphate Shunt, where it catalyzes the oxidation of Glucose-6-Phosphate to 6-Phosphogluconolactone. This is the first step in a process that results in formation of NADPH and eventually reduced glutathione (GSH).

G6PD deficiency is the most frequent inherited human enzyme defect in red blood cells. G6PD is an X-linked enzyme. Mutations in the G6PD gene result in different levels of enzyme activity, consequently causing a wide range of biochemical and clinical phenotypes [1,2]. Up to 140 different mutations have been identified in different parts of the world, each resulting in varying degrees of enzyme deficiency [1,3]. The World Health Organization has defined the different G6PD variants according to the enzyme levels, genotype and the severity of hemolysis. The clinical expression of G6PD deficiency varies from severe enzyme deficiency to increased enzyme activity (class I to class V). The commonest clinical patterns are; 1) neonatal jaundice, 2) congenital hemolytic anemia, 3) drug-induced hemolysis and 4) favism [1,2].

It is estimated that up to 400 million people worldwide have this deficiency. The high incidence of G6PD deficiency in Africa is likely due to malaria. The striking geographic correlation of G6PD deficiency distribution with the historical endemicity patterns of malaria has led to suggestions that two are linked. Studies to confirm this link have been equivocal [4-10]; this might be due to the different methods and diagnostic tests. Most studies in Africa show that the two X-linked mutations A376G and G202A cause between 85-100% of G6PD deficiency seen in Africa, and that both mutations have to be present for the enzyme deficiency to occur. In this study the G202A mutation was assessed for [9,11].

This was a sub-study of an ongoing cohort study of children for potential malaria vaccine trials. The objective of this sub-study was to determine the prevalence of G6PD deficiency and determine its relationship with *P. falciparum* infection prevalence (asymptomatic infection), incidence and parasite density.

## Methods

This study was a longitudinal study conducted starting October 1st 2008 to 31st December 2009. Field methods:

A total of 245 children between 6 months and 9 years where recruited by stratified random sampling from Iganga district in eastern Uganda. Majority of the inhabitants are from the ethnic Basoga tribe. Only one child per household was recruited to minimize clustering and if there was more than one potential subject in the household then a ballot was used. After informed consent, blood samples were collected. The children were followed up for 1 year with 114 and 135 in active and passive arms respectively. Allocation to each arm was done randomly. Those in the active arm were visited at home fortnightly and had a blood smear and body temperature taken on each visit. They were also asked to report to the study clinic if any illness occurred. Those in passive arm were asked to report to the study clinic in case of any illness. All study participants who developed malaria were treated with Artemether/lumefantrine in standard doses as per Uganda treatment guidelines.

### Laboratory methods

DNA was analyzed for the presence for the presence of one of the common G6PD mutations G → A at nt 202 using Polymerase chain reaction-restriction fragment length polymorphism (PCR-RFLP) method. DNA was extracted from a prepared buffy coat using E.Z.N.A blood DNA kits (Omega Bio-Tek Inc. Doraville. GA 30362. USA). PCR amplification was done using primers F – 5’-CCA CCA CTG CCC CTG TGA CCT-3’ and R- 5’-GGC CCT GAC ACCACC CAC CTT-3’. Details of the PCR-RFLP process are described elsewhere [9].

Thick blood smears were done to assess for malaria parasites using equal volumes of venous blood and stained with 2% Giemsa. All the blood smears were then double read independently by trained laboratory technicians. Where results between the two expert microscopists were discordant, a third microscopist re-read the blood slide and the majority decision was accepted as the final result. Parasite densities were determined from thick blood smears by examining the smears under × 100 objective. The average number of parasites/oil immersion field was then determined and the parasite density in number/μl was calculated by multiplying the average number of parasites per field by a microscope factor of 1/0.002. Samples collected at baseline were used to determine prevalence and G6PD status of the participants.

### Data analysis

STATA statistical software version 10.0 (StataCorp, College Station, TX) was used for analysis. Descriptive statistics were expressed as proportions and means ± SD (Range). For the relationship between G6PD status and P. falciparum infection, two by two tables were used to calculate Chi squared tests and odds ratio with 95% confidence intervals. The t test for unequal variances was used for comparison between malaria parasite density and different genotypes. Spearman’s correlation coefficient was used to test the relationship between parasite density and age. Incidence rates and rate ratios were calculated from the follow up data. A p-value < 0.05 was taken as statistically significant.

Study was approved by the School of Medicine, Research and Ethics Committee of Makerere College of Health Sciences and Uganda National Council for Science.

## Results

**Figure 1:**
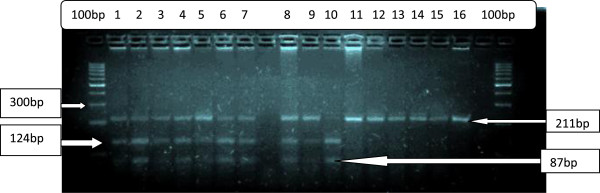
**Showing the final product after PCR-RFLP gel electrophoresis.** The photo shows the molecular marker 100 bp column bands, 211 bp bands for wild type; 211 bp, 124 bp and 87 bp bands for heterozygous samples and 87 bp and 124 bp for homozygous samples. The columns 12 and 16 had the control samples.

A total of two hundred forty five (245) children were recruited into the study. Of the children approached, 97.5% of those eligible were enrolled. One hundred and fourteen subjects (46.5%) were male. The age ranged from six (6) months to nine (9) years with a mean age of 4.4 (±2.3) years. The majority 232 (95%) of the study population were of the Basoga ethnic group which is the predominant tribe in Iganga. Ninety two percent (225/245) of the study participants reported to have used an Insecticide Treated Net in the night preceding the interview. The average household size was five inhabitants. Of the children recruited into the study the median family position was third. Fifteen percent (20/134) did not complete the anticipated follow up period of one year due to migration out of the study site. Overall twenty four percent (50/245) of the study participants had a G6PD A-mutation. The distribution of the prevalence of G6PD A genotypes among the study participants are shown in Table 1.Overall among those with the mutation, heterozygous females were highest at 12.6% (31/245) prevalence followed by hemizygous males 6.6% (16/303) and lowest was homozygous females 1.2% (3/245) Figure 1.

**Table 1:** Showing overall prevalence of G6PD A- genotypes among the 245 study children

**GENOTYPE/SEX**	**WILD TYPE**	**HETEROZYGOUS**	**HOMO/HEMIZYGOUS**
Overall	195 (79.59%)	31 (12.65%)	19 (7.76%)
Males	98 (86%)	NA	16 (14%)
Females	97 (74%)	31 (23.7%)	3 (2.3%)

This prevalence of asymptomatic P falciparum infection was 40.8% (100 of 245) among the children recruited at the baseline. The P. falciparum gametocyte carriage rate was 22% (55/245) among the study participants at baseline Table 2.

**Table 2:** **Table showing baseline thick blood smear results for *P. falciparum *** **trophozoites and gametocytes**

**Malaria blood smear result**	**Frequency (%)**
Negative for trophozoites	145 (59.2%)
Positive for trophozoites	100 (40.8%)
Positive for Gametocytes*	55 (22.5%)

*Note: 2 participants had gametocytes without trophozoites.

During the one year follow up, a total of 64 cumulative malaria episodes of symptomatic malaria were recorded among the 245 study participants. 55.4% (31/56) had only one malaria episode, 28.5% (16/56) had two, 12.5% (7/56) suffered three episodes while 3.6% (2/56) had four malaria episodes during the one year follow up period.

The relationship between G6PA mutations and presence of malaria parasites was assessed for by comparing proportions using chi square test. There was a lower prevalence of *P. falciparum* infection among the G6PD A- heterozygous females compared to the wild type and homozygous but this was not statistically significant; 29.03%, 42.6%, and 43% respectively OR 0.55, (95% CI 0.24- 1.24, p = 0.11). The percentage of G6PD A hemizygous males harboring malaria parasites was not statistically different from those with the wild type G6PD (42.6% versus 42.1%, OR 0.98 (95% CI 0.37 – 2.55), p = 0.9.

Malaria incidence rates were 0.56 episodes/year for G6PD wild-type, and 0.52 episodes/year for G6PD A - female heterozygote’s (p = 0.5) and there was only 1 homozygous male followed up actively. Of the children who had *P. falciparum* infection, the geometric mean parasite density was 2285 (range 250 – 41150) parasites/μl. There was a statistically significant difference in mean parasite densities between the wild types and the heterozygote’s, with lower parasite densities among the female G6PD A- heterozygote’s compared to wild type with; 941 parasites/μl versus 2505 parasites/μl respectively with P = 0.024, using a t test with unequal variances). There was a weak negative correlation between malaria parasite density and age, with r = - 0.33 (p = 0.0008).

## Discussion

It is now apparent that genetic influence of malaria susceptibility in humans is very important. Understanding these interactions may influence the different interventions especially with drug therapy for malaria. This is important in clinical trials especially vaccine trials as this would impact on the outcome endpoints. The prevalence of G6PD was not known in our study site.

This study reveals that the prevalence of the G6PD A- genotype is high in this part of Uganda at 20.4%. This rate is within the range of 15-30% that has been found elsewhere in sub-Saharan Africa [5,12-14] and comparable to that found in Uganda, although the populations were of different ethnicity [9]. This high prevalence raises concern and need for caution within this study population when using drugs which cause oxidative stress on RBCs such as Primaquine or other compounds that can spark off hemoglobinuria.

Of the 245 children studied, 40.82% were found to have asymptomatic *P falciparum* infection. This rate however should be interpreted in context of the period of recruitment that coincided with a period of prolonged dry weather as this would result in lower infection rates. The rate of asymptomatic *P. falciparum* infections suggests that this is an area of moderate malaria transmission. Higher asymptomatic *P. falciparum* infection rates of greater than 70% are seen in areas of high transmission and rates of lower than 10% are seen in areas of low transmission [11,15-17]. Asymptomatic *P falciparum* infection probably plays an important role in maintaining malaria endemicity by providing a continuous source of parasites that helps perpetuate malaria transmission [16,17]. In addition it may be associated with anemia in infected children and more morbidity in those with sickle cell anemia [15,18,19]. The only possible benefit is that asymptomatic *P falciparum* infection might play an important role in the development of malaria specific immunity [19-21].

The lack of a significant difference in the both prevalence and incidence rates of infection among the different G6PD genotypes suggests this mutation might not influence susceptibility to malaria. On the other hand, the significant difference in parasite densities among the heterozygous females and the wild types points towards the fact that this mutation might influence disease severity as parasite density is a known factor in disease severity. This is in keeping with longitudinal studies on the relationship between malaria and G6PD polymorphisms that have shown that G6PD A- individuals have lower incidence of severe malaria [8,9,14]. These findings support the hypothesis that differences in malaria severity among the different G6PD polymorphisms is due to the impact of G6PD status on parasite densities [20-23]. The findings on differences in prevalence is not consistent with the a similar study conducted in Gabon that showed that female heterozygous children were protected against asymptomatic malaria with rates of 39% versus 67% (p = 0.03) compared to those with wild type G6PD alleles [13]. However in the Gabonese study, the more sensitive PCR was used to diagnose asymptomatic infection and the participant age group was 7 to 19 years while the children were followed up for 4 days to exclude later development of symptoms. We did not find a significant difference in incidence rates in this population among the different G6PD polymorphisms. Considering that other studies have showed that numerous other genetic polymorphisms have an association with malaria including; sickle cell trait and disease, blood group, nitric oxide synthase, Tumor Necrosis Factor (TNF) α promoter, haptoglobin, α-thalasemia, intercellular adhesion molecule–1, and RANTES, the interactions involving all these genotypes may be complex. This might necessitate a large longitudinal study to further clarify on the biological and statistical interactions of all these genetic polymorphisms with malaria susceptibility [3,9,12,13,22].

Limitations of this study include; Use of microscopy to determine *P falciparum* asymptomatic infection could have resulted in a lower prevalence as a PCR assay would have been more accurate for this and only one host factor that influences susceptibility to malaria i.e. G6PD status was studied hence no multivariate analysis was done.

## Conclusions

This study showed that 20.41% of the population in this part of Uganda carry the G6PD A-mutation, within the range of 15-32% seen in other parts of Africa. *P. falciparum* infection incidence and prevalence rates are similar among the G6PD genotypes though, once infected, *P. falciparum* parasite densities are lowest among G6PD A- heterozygous females. This suggests differences in *P. falciparum* infection rates and severity of disease could be mediated by differences in parasite densities among the different G6PD genotypes. Given this high prevalence of the G6PD A- mutation, screening for this mutation is warranted in the work up of patients with acute hemolytic anemia in this population. It has also shown that up to 40% of otherwise healthy children harbor malaria parasites. It is likely that the interaction between malaria and these polymorphisms is complex thus a clear impact for one of them might not be easily discernible from a single cross sectional study. A large longitudinal study to assess the role and interaction of each of these is necessary.

## Abbreviations

DNA: Deoxyribonucleic acid; G202A: Mutation in the G6PD gene with exchange of guanine for adenine at position 202; GSH: Reduced glutathione; G6PD: Glucose-6-phosphate dehydrogenate; NADPH: Reduced nicotinamide adenine dinucleotide phosphate; PCR: Polymerase chain reaction; RFLP: Restriction fragmentation length polymorphism; TNF: Tumor Necrosis factor.

## Competing interests

We declare that we have no competing interests.

## Authors’ contributions

BD: Participated in participant recruitment and conducted the G6PD assays. HD and KM participated in the study conception and supervision of the study. FK Conceived the study supervised the data collection and ensured quality of the laboratory results. All authors participated in the writing and review of the manuscript. All authors read and approved the final manuscript.
